# When Biology Gets Personal: Hidden Challenges of Privacy and Ethics in Biological Big Data

**Published:** 2019

**Authors:** Gamze Gürsoy, Arif Harmanci, Haixu Tang, Erman Ayday, Steven E. Brenner

**Affiliations:** Computational Biology and Bioinformatics Program, Molecular Biophysics & Biochemistry, Yale University, New Haven, CT, 06511, USA; Center for Precision Health, School of Biomedical Informatics, University of Texas Health Science Center, Houston, TX, 77030, USA; School of Informatics, Computing and Engineering, Indiana University Bloomington, Bloomington, IN, 47405, USA; Department of Electrical Engineering and Computer Science, Case Western Reserve University, Cleveland, OH, 44106, USA; University of California Berkeley, CA, 94720-3012, USA

**Keywords:** biological data privacy, genomics, genetic testing

## Abstract

High-throughput technologies for biological data acquisition are advancing at an increasing pace. Most prominently, the decreasing cost of DNA sequencing has led to an exponential growth of sequence information, including individual human genomes. This session of the 2019 Pacific Symposium on Biocomputing presents the distinctive privacy and ethical challenges related to the generation, storage, processing, study, and sharing of individuals’ biological data generated by multitude of technologies including but not limited to genomics, proteomics, metagenomics, bioimaging, biosensors, and personal health trackers. The mission is to bring together computational biologists, experimental biologists, computer scientists, ethicists, and policy and lawmakers to share ideas, discuss the challenges related to biological data and privacy.

## 1. Introduction

Data privacy is an important topic of debate crossing many different fields such as ethics, sociology, law, political science and forensic science. Thanks to the rapid reduction of the DNA sequencing cost in the past decade, the number and the volume of available genomic data have exponentially increased [1]. Hence, individuals’ genomic data has recently emerged as one of the major foci of studies on privacy as availability of genetic information gives rise to privacy concerns [2]. For example, individuals express concern that genetic predisposition to diseases may bias insurance companies or enable unlawful discrimination by employers [3,4,5]. On a larger scale, imagine the economic repercussions had it been leaked that the CEO of Apple Computer had pancreatic cancer and was not adhering to a typical oncological regimen. Recently it has been also shown that high throughput molecular phenotype datasets such as functional genomic and metabolomics measurements, and microbiome measurements increased the number of quasi-identifiers for participating individuals that can be used by adversaries for re-identification purposes [6,7,8,9]. In addition, the emergence of electronic health records (EHR) with the rise of personalized medicine makes patients vulnerable to breaching privacy. These results indicate that privacy concerns over sharing personalized biological data will increase quickly with the increase in the number of genetic and ancestry testing companies, which collect and distribute very large amount of health related data, including genetic data (such as 23andMe) or health and fitness tracking data (such as fitbit). The data collection and sharing methods that these companies use call for a public discussion of privacy considerations around these new concepts. Moreover, the recent arrest of the Golden State Killer, through long-range familial search on consumer genomics databases, sparked questions over the risk of re-identification based on genetic testing taken by a relative. Recent two studies showed the statistical risk of identifying relatives as being high by using long-range familial searches [10,11].

Protecting the privacy of study participants has emerged as an important issue in genotype-phenotype association studies. Several studies investigated whether a genome of an individual can be detected in a mixture [12,13,14,15]. As a result, various counter-measures have been proposed to protect participant privacy [16]. As the number of genotype-phenotype datasets increase, new routes for breaching privacy such as cross-referencing multiple databases opened up [17,18]. Access control, data anonymization and cryptographic techniques were studied to prevent privacy breaches [4]. Ultimately, the ability to keep these data private is unclear, and so preparations for both small and catastrophic leaks must be made [5]. As the technologies increase, new data types are being released and more studies to investigate the potential privacy breaches will be needed. This area of research has become more and more interdisciplinary, where ethics researchers inform researchers who work on privacy-preserving techniques, while these techniques inform policymakers to reform laws and policies.

On the other side of the privacy problem, the benefit and importance of open data sharing is widely acknowledged, as the solutions such as access control or cryptographic techniques delay the access to the data by average researchers either by creating bureaucratic bottlenecks or technical challenges. Open data sharing harbors the collaboration between different biomedical researchers by allowing rapid exchange of the information. Funding agencies and research organizations are increasingly supporting new means of data sharing and new requirements for making data publicly available while preserving participants’ privacy [19]. This increases the value of the techniques and policies that prevent the sensitive information leakage while promoting data sharing.

The papers featured in this session represent various aspects of biological data privacy highlighting a number of problems and solutions that need to be addressed to protect privacy of individuals while encouraging open data sharing. Topics in this session include making inferences on complex phenotypes in large biobanks, patient re-identification through electronic health records and countermeasures, privacy-preserving GWAS studies as well as efforts on improving informed consents for AllofUs research project.

## 2. Podium Presentations

After the seminal work by Homer et al. [12], the policies on how to share GWAS results have been changed and only summary statistics are allowed to share publicly. **Gasdaska et al.** [20] explore the possibility of using these summary statistics to make inferences about the hidden, complex phenotypes that are derived from two or more phenotypes. This potentially reveals information about the participants that they may not want to disclose. Investigators validated their statistical derivations on simulated and real datasets.

As **A. Gasdaska** and colleagues [20] showed that sharing statistical aggregates from GWAS might have sensitive information leakages and also demonstrated that how complex phenotypes can be analyzed in terms of simple phenotypes in a privacy preserving fashion, **S. Simmons** and colleagues [21] showed us how we could reduce this leakage by introducing a Laplacian noise to the released data. The investigators presented a novel method for measuring privacy loss in GWAS summary statistics. This was achieved by providing a probabilistic formulation for measuring the risk of releasing summary statistics as the posterior probability of an individual being in the cohort. With the introduction of an MCMC-based method for computing this posterior probability, the authors reduced the degree of privacy leakage with the same amount of data released. This work presented interesting ideas on how to control the privacy risk by setting a noise level and the amount of data to be released.

**K. Johnson** and colleagues [22] studied the privacy leakages of Electronic Health Records. They showed that lab tests can be used as quasi-identifiers for patients for re-identification of patients’ medical records. The investigators used the EHR at Mount Sinai Hospital as a case study. This study took an even more interesting turn when they used variational auto-encoder to encode the lab test results to reduce the privacy risk of re-identification. They showed a substantial decrease in re-identification risks when the lab tests were stored as latent variables while the encoded test results still provide almost the same utility as original results when compared in terms of classification accuracy. Although further work is required to show how decoding-encoding will be achieved in this new representation, the novel idea of storing data will open up the doors for storing other kind of private data in the future.

## 3. Posters with Published Papers

This year’s poster session with papers published in the proceedings will feature a unique study that has not been explored at PSB before by **M. Doerr** and colleagues [23]. They designed a study to give a comprehensive overview of existing jurisdictions for the informed consent process for the AllofUs initiative and its compliance with the state/territory regulations. This study will be of great interest for the investigators of the AllofUs project, which aims to collect a vast amount of biomedical data from a million of Americans.
